# Trends of secondhand smoke exposure among children: A scientometric analysis

**DOI:** 10.18332/tid/202017

**Published:** 2025-03-20

**Authors:** Hong Lu, Shaojie Qi, Wenqi Chen, Dingge Gu

**Affiliations:** 1 Research Institute of Social Development, Southwestern University of Finance and Economics, Chengdu, China; 2 School of Management Science and Engineering, Southwestern University of Finance and Economics, Chengdu, China

**Keywords:** secondhand smoke, children, scientometric analysis

## Abstract

**INTRODUCTION:**

Secondhand smoke (SHS) exposure poses significant risks to children’s health, yet global research on this issue requires further analysis to facilitate a comprehensive exploration of knowledge production and topic trends. This study aims to analyze the research outputs, cooperation and evolution on children’s exposure to secondhand smoke.

**METHODS:**

A scientometric approach was employed to analyze global research on adolescent secondhand smoke exposure. Data were gathered from scholarly databases and analyzed using CiteSpace software, which was used to assess publication trends, citation patterns, and research collaborations. The study examined publications, citations, interdisciplinary collaboration across countries, institutions, authors, and disciplines, as well as identifying research hotspots and emerging trends using burst detection and co-citation network analysis.

**RESULTS:**

The analysis revealed significant growth in research on adolescent SHS exposure over recent decades, with the United States, United Kingdom, and China being the most productive countries in this field. Key journals in the substance abuse field serve as the primary venues for publishing relevant studies. Interdisciplinary collaborations have increased, particularly between health, policy, and social science disciplines. Research hotspots include the impact of SHS on children’s physical and mental health, with a particular focus on behavioral and developmental issues. Additionally, e-cigarette and heated tobacco products have emerged as new concerns in recent research.

**CONCLUSIONS:**

The study highlights the rapid expansion of research on SHS exposure among children, underscoring the growing recognition of its widespread health impacts. Interdisciplinary research collaborations are becoming more prevalent, and significant efforts are needed to address emerging issues such as e-cigarette exposure. These results underscore the need for further research to explore and address these evolving issues.

## INTRODUCTION

Secondhand smoke (SHS) has emerged as a major public health concern threatening global health^1^. The World Health Organization (WHO) defines secondhand smoke as the smoke that permeates enclosed spaces when individuals burn tobacco products, including cigarettes, hookahs, and others^2^. Although secondhand smoke exposure poses health risks across all age groups, children and adolescents are especially vulnerable^3^. The 2019 World Health Organization report estimates that secondhand smoke causes approximately 1.2 million deaths annually worldwide, including around 65000 deaths among children and adolescents^4^. Secondhand smoke exposure is a leading cause of disease in children and is linked to asthma, otitis media, lower respiratory infections, and sudden infant death syndrome^5^. Additionally, secondhand smoke exposure during pregnancy has been shown to result in preterm labor, low birth weight, small for gestational age infant, and perinatal mortality^6^. Furthermore, children exposed to secondhand smoke are more likely to initiate their own tobacco use^7^. Studies have also shown that secondhand smoke increases the risk of neurological disorders by 50%, including learning disabilities, depression, and behavioral problems^8^.

Children and adolescents are in a critical period of growth and development, during which their organs and tissues are more sensitive to toxic and harmful substances. As a result, tobacco control measures for children and adolescents have been implemented by WHO, governments, and the education sector^4^. Over the past two decades, the enforcement of smoking bans in public places has indirectly led to a higher prevalence of voluntary smoking bans in homes, which has contributed to a reduction in SHS-related diseases among children and infants^9^. Nevertheless, in many countries worldwide, children and adolescents continue to be exposed to secondhand smoke^10^.

In the EU, the rate of children exposed to secondhand smoke within the home has been shown to exceed 10%. Among pregnant women, this rate remained at approximately 20% from 2006 to 2017^11^. A recent global study revealed that, while adolescent household secondhand smoke exposure declined in most countries between 1999 and 2018, adolescent public secondhand smoke exposure either remained unchanged or showed a tendency to increase^12^. Additionally, the emergence of e-cigarettes and heated tobacco products (HTPs) since the 2010s has introduced a new potential source of hazardous indoor substances^13^. This escalating trend is concerning and may pose new challenges to children’s health and well-being.

While traditional review studies have contributed to understanding children’s secondhand smoke exposure^9,10,14^, their limitations in literature selection subjectivity and analytical scope restriction would impede holistic mapping of research trajectories. To address these gaps, this study employs scientometric analysis, a quantitative methodology leveraging statistical and computational techniques to explore various aspects of scientific activity and are widely used to assess longitudinal research outputs, analyze knowledge structures, and study disciplinary evolution. In this study, a scientometric analysis was conducted using a substantial bibliometric database to map the structure of knowledge and trends in secondhand smoke exposure among children. Specifically, several important questions will be answered: 1) ‘What are the overall changes and developments in the field?’; 2) ‘Which countries, institutions, and authors have been the most productive and influential?’; and 3) ‘What are the past and current research hotspots in the field, and how might future trends evolve?’.

## METHODS

### Search strategy

The data for this study were obtained from the Web of Science Core Collection, which includes high-quality academic literature. It is one of the most commonly used data sources in scientometric research^15^. Following the Preferred Reporting Items for Systematic Reviews and Meta-Analyses (PRISMA) guidelines, the retrieved records were screened and cleaned to ensure rigor and transparency (Supplementary file Figure 1)^16^. Only peer-reviewed articles and review articles were included, while non-research documents (e.g. meeting abstracts, editorials) were excluded to ensure analytical rigor. The specific search terms and inclusion criteria are detailed in Supplementary file Table 1. The initial search recorded 5664 documents, which were independently screened by two researchers based on article titles and abstracts. Disagreements were resolved through discussions, resulting in 1695 documents for analysis (data retrieved on 1 December 2024).

**Table 1:** Top 10 most productive journals (1984–2024)

*Journal*	*Categories*	*Count*	*Impact factor (2023)*
Nicotine & Tobacco Research	Public, Environmental & Occupational Health	71	3.00
International Journal of Environmental Research and Public Health	Public, Environmental & Occupational Health	60	4.61 (2021)
Pediatrics	Pediatrics	44	6.20
Tobacco Control	Public, Environmental & Occupational Health	42	4.00
Tobacco Induced Diseases	Public, Environmental & Occupational Health	33	2.20
BMC Public Health	Public, Environmental & Occupational Health	32	3.50
Preventive Medicine	Medicine, General & Internal	28	4.30
Journal of Asthma	Allergy	23	1.70
Plos One	Multidisciplinary Sciences	23	2.90
Chest	Critical Care Medicine	20	9.50

### Analysis plan

Specifically, the present study first analyzed the trends in the number of publications (NP) and the number of citations (NC) in adolescent secondhand smoke research over time and summarized the most productive journals. Second, research collaborations in this field were examined across four dimensions: country, institution, author, and discipline. To assess the contributions of these entities, the Betweenness Centrality (BC) index was applied to quantify the bridging role of nodes within the collaboration network^17^. Nodes with a BC index exceeding 0.1 or marked by a purple outer ring are classified as high-BC nodes, indicating significant collaborative contributions^18^. Productive authors were identified based on Lotka and Price Law^19^, with the formula as follows:


$$\text{m}=0.749\times \sqrt{{\text{n}}_{\max}}$$

where n_max_ represents the publication count of the most prolific authors.

Finally, burst detection analysis and co-citation network analysis were employed to identify hotspots and trends in adolescent secondhand smoke research. Burst detection identifies sudden keyword frequency surges in specific time windows to detect emerging trends. Co-citation analysis maps disciplinary knowledge structures by measuring how frequently two documents are cited together^20^. The scientometric analysis in this study was conducted primarily using CiteSpace, a software tool developed by Chen^21^ at Drexel University College of Computing and Informatics. This software assists researchers in analyzing publication and citation patterns, thereby enhancing understanding of scientific output, disciplinary evolution, and research frontiers^20^.

## RESULTS

### Publications and main journals

The scientometric analysis of adolescent secondhand smoke research revealed a significant increase in both the number of publications and citations over time. Supplementary file Figure 2 presents the number of publications (NP) and number of citations (NC) from 1984 to 2024. It is clear that the number of publications has steadily increased, particularly after 2000. The number of publications peaked at 126 in 2024, marking a significant increase in recent years. The citation data shows a different upward trend, with a peak citation count of 2628 in 2003. In recent years, although the number of publications remained relatively high, citation counts dropped significantly.

The analysis of the most productive journals in the field revealed that research on secondhand smoke and its effects on children and adolescents has been published in various journals across multiple disciplines. The top 10 most productive journals, listed in Table 1, reflect the interdisciplinary nature of the topic, spanning public health, pediatrics, tobacco control, and respiratory medicine. The journal *Nicotine & Tobacco Research* was the most productive, contributing 71 articles, followed by *International Journal of Environmental Research and Public Health* with 60 articles. Other notable journals include *Pediatrics* (n=44), *Tobacco Control* (n=42), and *Tobacco Induced Diseases* (n=33). These journals are primarily focused on public, environmental, and occupational health, which aligns with the central concerns of secondhand smoke research. Additionally, research in this field is widely published in high-impact journals.

Based on the analysis, the studies published in these journals have concentrated on three areas: health risks, exposure assessment, and interventions. Research on health risks predominantly examines the impact of secondhand smoke on respiratory diseases and neurobehavioral issues. Exposure assessment studies primarily aim to measure secondhand smoke exposure, using biomarkers quantitative markers, in conjunction with environmental monitoring in both household and public settings. Intervention research, although limited in number, has notably increased since 2015, with a particular focus on evaluating the effectiveness of no-smoking interventions within the home and public spaces. Methodologically, longitudinal studies have provided significant insights into the long-term effects of secondhand smoke exposure, including its association with asthma, cerebral palsy, hearing impairments, and offspring smoking behavior. Additionally, these studies have contributed valuable knowledge on the effectiveness of health interventions in both school and home environments.

### Collaboration network of adolescent secondhand smoke research

*Country and region collaboration networks*

**Figure 1:**
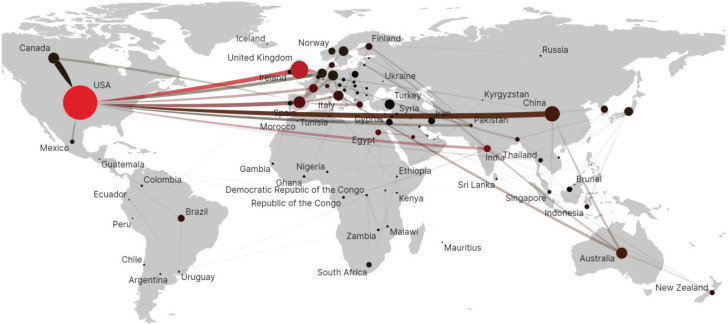
Country or region collaboration network from 1984 to 2024. Each node represents a country, and the size of the node the number of publications of the country. Each edge indicates a collaborative relationship between countries (N=1695)

The collaboration network analysis revealed that 89 countries and regions have contributed to research on secondhand smoke and its impact on children and adolescents (Table 2). The United States was the leading country, with 713 publications and a BC index of 0.53, indicating its central role in the collaboration network. Following were England (n=190, BC=0.34), China (n=142, BC=0.05), Spain (n=84, BC=0.06), Australia (n=76, BC=0.05), and Canada (n=73, BC=0.02), which also made notable contributions to the field. Several countries from other regions, including India, South Korea, and Brazil, have also made significant contributions to the literature, with India showing a particularly high BC index of 0.13, reflecting its rising influence in the research network since 2001. Overall, the results highlight the diverse global participation in research on secondhand smoke and its impact on children and adolescents, with some countries playing a more central role in the collaboration network (Figure 1).

**Table 2:** Top 10 most productive countries and institutions (1984–2024)

*Country*	*Count*	*BC*	*Year*	*Institution*	*Count*	*BC*	*Year*
USA	713	0.53	1984	University System of Ohio	81	0.13	1996
UK	190	0.34	1991	University of California System	80	0.06	1987
China	142	0.05	1988	Harvard University	79	0.27	1984
Spain	84	0.06	1993	Cincinnati Children’s Hospital Medical Center	59	0.04	2003
Australia	76	0.05	1993	University of Cincinnati	56	0.02	2003
Canada	73	0.02	1988	Johns Hopkins University	56	0.04	1997
Italy	62	0.03	1989	California State University System	48	0.02	1997
Germany	61	0.02	1991	San Diego State University	46	0.02	1997
Sweden	56	0.02	1988	Centers for Disease Control and Prevention (CDC)	41	0.15	1991
Netherlands	52	0.02	1996	Centro de Investigación Biomédica en Red (CIBER)	41	0.03	2009

BC: Betweenness Centrality.

*Institution collaboration networks*

**Figure 2:**
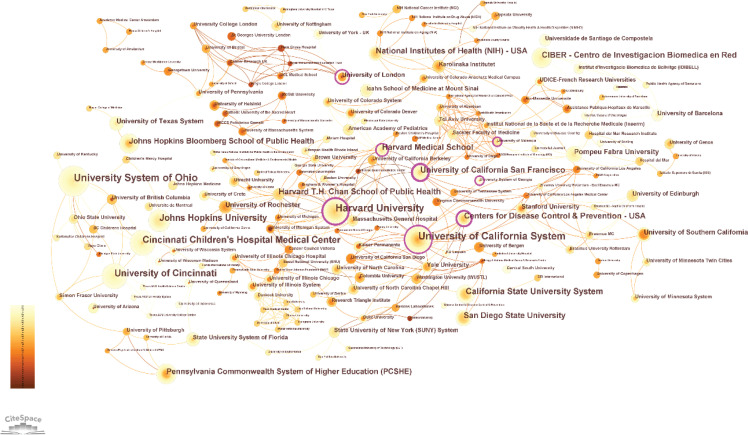
Institution collaboration network from 1984 to 2024. Each node represents an institution, and the size of the node indicates the number of publications of the institution. Each edge indicates a collaborative relationship between two institutions (N=1695)

As shown in Table 2, the University System of Ohio, with 81 publications and a BC index of 0.13, was the most productive institution in this field. Harvard University closely followed, with 79 publications and a BC index of 0.27, indicating its central position in the collaboration network. These institutions were key nodes, with their relatively high BC indices indicating substantial collaborative contributions. Other prominent institutions include the University of California System (n=80, BC=0.06) and Cincinnati Children’s Hospital Medical Center (n=59, BC=0.04). The Centers for Disease Control and Prevention (CDC), with 41 publications and a BC index of 0.15, also emerged as a significant contributor, reflecting its role in public health research. Overall, the institutional collaboration network highlights the significant role played by major universities and research centers, particularly in the United States, in shaping the direction and output of research on the health effects of secondhand smoke on younger populations (Figure 2).

*Author collaboration network*

**Figure 3:**
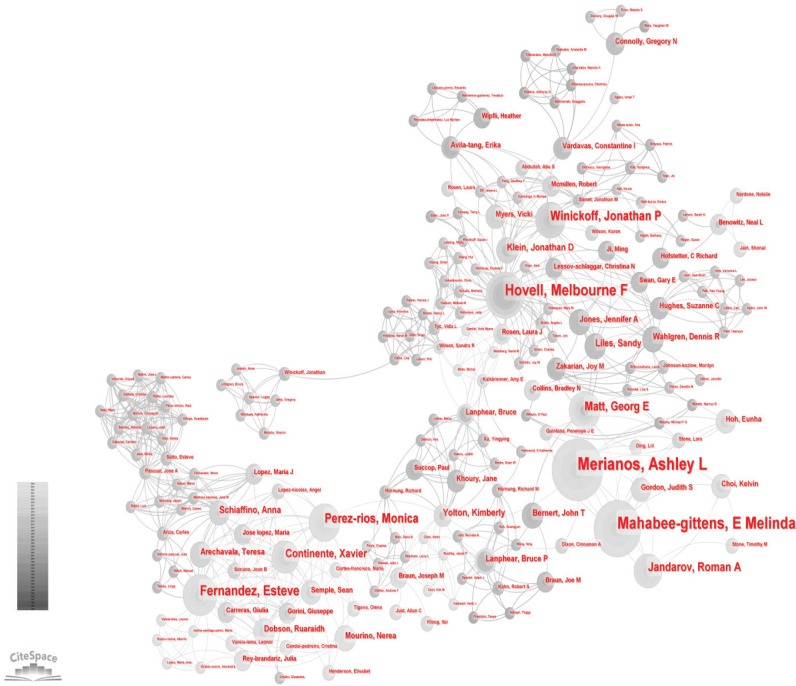
Author collaboration network from 1984 to 2024. Each node represents an author, and the size of the node indicates the number of publications of the author. Each edge indicates a collaborative relationship between two authors (N=1695)

From 1984 to 2024, there were 185 most productive authors, we list the top 11 core authors in Table 3. Ashley L. Merianos and E. Melinda Mahabee-Gittens were the most productive authors, with 35 and 33 papers, respectively. However, there is no evident BC index. Other significant contributors include Melbourne F. Hovell (n=24, BC=0.02), Esteve Fernandez (n=18, BC=0), and Jonathan P. Winickoff (n=16, BC=0). Monica Perez-Rios, Georg E. Matt, and Roman A. Jandarov, each with ≥15 publications, who were also among the top authors in the field. However, none of these authors had a Betweenness Centrality (BC) index exceeding 0.1. This suggests that, while these authors have published extensively, they were not closely connected (Figure 3).

**Table 3:** Top 11 most productive authors (1984–2024)

*Author*	*BC*	*Count*	*Year*
Merianos, Ashley L.	0	35	2016
Mahabee-Gittens, E. Melinda	0	33	2016
Hovell, Melbourne F.	0.02	24	2007
Fernandez, Esteve	0	18	2009
Winickoff, Jonathan P.	0	16	2011
Perez-Rios, Monica	0	15	2018
Matt, Georg E.	0	15	2008
Jandarov, Roman A.	0	12	2017
Continente, Xavier	0	11	2018
Yolton, Kimberly	0	10	2008
Rosen, Laura J.	0	10	2011

BC: Betweenness Centrality.

*Category collaboration network*

**Figure 4:**
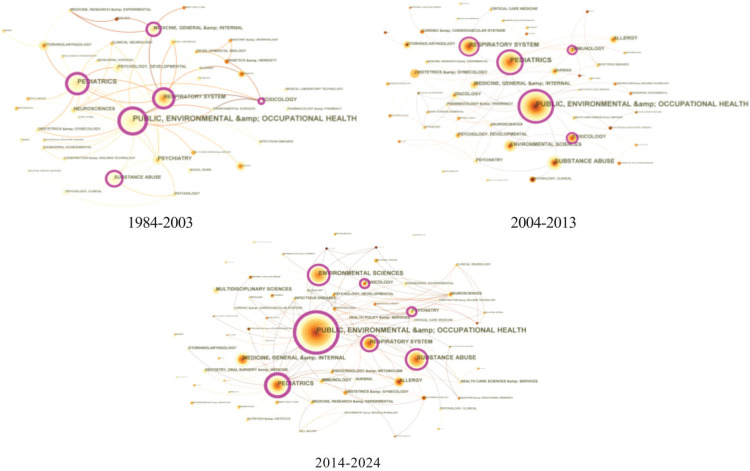
Category collaboration network from 1984 to 2024. Each node represents a category, and the size of the node corresponds to the number of articles published. Each edge signifies a collaborative relationship between two categories (N=1695)

The analysis of the disciplinary collaboration network was conducted over three distinct time periods, over which it evolved significantly. From 1984 to 2003, research primarily focused on Public, Environmental, and Occupational Health, with limited cross-disciplinary interaction. Between 2004 and 2013, collaboration expanded, with Pediatrics and Tobacco Control becoming more prominent, reflecting a broader recognition of the issue’s complexity. From 2014 to 2024, there was further growth, with increased interdisciplinary collaboration, particularly between Pediatrics, Tobacco Control, and emerging fields such as Allergy and Critical Care Medicine. This trend indicates a growing understanding of the health impacts of secondhand smoke and the increasing need for multi-disciplinary approaches to address the issue (Figure 4).

### Research hotspots and trends

*Keyword co-occurrence network*

The keyword co-occurrence network analysis provides insights into the central themes in research on secondhand smoke exposure in children and adolescents. As shown in Supplementary file Figure 3, terms such as ‘secondhand smoke’, ‘children’, ‘adolescents’, and ‘health’ prominently appear, reflecting the core focus of the research. Over time, keywords related to specific health outcomes, such as ‘asthma’, ‘respiratory diseases’, and ‘behavioral effects’, have become more prominent, highlighting the growing understanding of the diverse impacts of secondhand smoke on children and adolescent health. Additionally, keywords such as ‘prevention’, ‘intervention’, and ‘policy’ have become more frequent in recent years, indicating an increasing focus on addressing the issue through actionable strategies and public health measures. This keyword map underscores the shift toward a more comprehensive approach, integrating both health impacts and policy solutions to mitigate the effects of secondhand smoke.

*Document co-citation*

Co-citation analysis is an effective tool for identifying and analyzing the core research topics and knowledge structure of a specific field. Supplementary file Figure 4 identifies several key research themes and clusters within the literature on secondhand smoke exposure.

The analysis revealed distinct clusters based on the primary focus of the studies, including health effects, exposure assessments, intervention strategies, and policy frameworks. The first category focused on the health impacts of secondhand smoke, particularly its effects on respiratory diseases and developmental outcomes in children and adolescents. Studies in this cluster often related to the pathophysiology of secondhand smoke exposure and its association with asthma, lung function, and cognitive development.

The second category concentrated on exposure assessments, with research focusing on measuring and quantifying secondhand smoke exposure in various settings, including homes, schools, and public spaces. This cluster also included studies on biomarkers and exposure monitoring techniques, emphasizing the importance of accurate assessment methods in understanding the scope of the problem.

The third category emerged around intervention strategies, including public health campaigns, smoking cessation programs, and regulatory measures to limit secondhand smoke exposure. This cluster also highlighted studies on the effectiveness of policy interventions, such as smoking bans and the implementation of smoke-free environments, in reducing exposure and improving health outcomes.

Finally, the analysis revealed a cluster focused on the social and behavioral aspects of secondhand smoke exposure, including studies on public attitudes, parental smoking behavior, and the role of socio-economic factors in shaping exposure patterns. This cluster underscores the importance of understanding the social context in addressing secondhand smoke exposure among children and adolescents.

## DISCUSSION

Children have the right to grow up in a safe environment, and both parents and the government have a moral obligation to ensure this. Passive smoking poses a significant threat to children’s health. However, this issue has been relatively neglected in discussions about smoking, and it is now time to prioritize the health and rights of children by ensuring they grow up in smoke-free environments. A substantial body of evidence indicates that exposure to secondhand smoke severely harms both the physical and mental health of children^5^. Although academic interest in secondhand smoke exposure among children has grown since the early 2000s, it is important to acknowledge that current research progress is still insufficient to address the complexity of the challenges children face.

Research conducted by scholars in Western developed countries, constitutes the majority of knowledge production in this field, indicating that secondhand smoke exposure among children in these countries has been more extensively studied^22^. In contrast, children in underdeveloped countries face more severe secondhand smoke exposure, and these nations lag behind Western developed countries in terms of anti-smoking policies and public awareness^23^. Although numerous studies on secondhand smoke exposure among children have been published in China, the issue remains a significant concern for Chinese children. Research has shown that 41.7% of households in China are exposed to secondhand smoke, with an average daily exposure time of 14.7 ± 14.6 min. Furthermore, children in households with older parents and lower levels of parental education are more likely to be exposed to secondhand smoke^24^. This highlights the need to not only discuss the issue at an academic level but also take concrete actions to reduce children’s exposure to secondhand smoke. Indeed, many developing countries have also recognized this issue. Co-citation analysis reveals that children in developing countries have become a focal point in secondhand smoke exposure research in recent years. For instance, a study estimated that approximately 507.74 million children under 15 years of age in 21 low- and middle-income countries are exposed to inhalable secondhand smoke at home, with children in China, India, Bangladesh, Indonesia, and the Philippines accounting for nearly 84.6% of those exposed^1^. However, tobacco control has been relatively overlooked in low- and middle-income countries, as indicated by a meta-analysis in Sub-Saharan Africa^25^. These studies provide evidence to support cross-national comparisons, particularly when examining trends across different socioeconomic contexts.

Through co-citation and trend analysis, we found that the impact of secondhand smoke exposure on children’s behavior and mental health has become a major focus of global research. Exposure to secondhand smoke not only harms children’s physical health but also affects their mental well-being. A meta-analysis of 18 studies indicated that children who are frequently exposed to secondhand smoke are more likely to experience symptoms of depression and anxiety^26^. Moreover, children’s physical and mental health are closely interconnected; without good mental health, optimal physical health cannot be achieved. In addition, several studies have shown that secondhand smoke exposure can lead to behavioral issues, such as hyperactivity and inattention. Recent research underscores the urgent need to address the issue of secondhand smoke exposure in children.

Furthermore, e-cigarettes have rapidly gained popularity worldwide, driven by their diverse flavors, appealing packaging, and limited regulation in marketing^27^. This surge in e-cigarette usage has introduced a new public health concern. Children are increasingly affected by secondhand smoke from e-cigarettes. Despite manufacturers claims that e-cigarettes do not release harmful gases, studies have shown that inhaling e-cigarette secondhand smoke can lead to lung and cardiovascular diseases^28^. The aggressive marketing strategies of e-cigarette manufacturers may further exacerbate children’s exposure, as the public often perceives e-cigarette smoke as harmless and odorless.

Despite significant progress in research, several gaps and limitations remain. First, intervention studies have shown limited progress. Most studies on children’s secondhand smoke exposure focus on reducing exposure in public places and homes through policies, social norms, and educational campaigns^29^. For example, some studies suggest that involving community health workers in providing interventions for smokers at home can effectively reduce children’s exposure to secondhand smoke^30^, which is crucial for minimizing health risks. However, as a vulnerable group at risk from secondhand smoke, children are an important target for anti-smoking interventions. The role of children’s perceptions of secondhand smoke risks, which is essential, has been largely overlooked in current studies^31^. Empowering children to express opposition to secondhand smoke, as well as encouraging parents to create smoke-free homes and schools, could significantly reduce exposure.

Second, research on secondhand smoke from e-cigarettes and heated tobacco products has advanced slowly. Current studies primarily focus on the composition of secondhand smoke from these products^32^. As e-cigarettes gain popularity, particularly for indoor use, it is important for policymakers to include e-cigarettes in smoke-free regulations to mitigate their impact on children. Given the rapid development and diversification of e-cigarette products, future research should focus more on secondhand smoke from e-cigarettes. Recent trends indicate that the proportion of adolescents exposed to secondhand smoke from e-cigarettes and heated tobacco products is rising^33^. However, there are no available statistics for children or adolescents, and research on global exposure to secondhand smoke from e-cigarettes and heated tobacco products remains scarce. This lack of data hinders the development of effective regulatory policies.

### Limitations

This study has several limitations. First, only English-language articles were included, which may restrict the analysis by excluding relevant research published in other languages. Second, this study did not incorporate books, conference reports, and other types of research publications. Future research should aim to include a broader range of languages and research formats. Third, this study focused solely on published articles, potentially overlooking valuable unpublished research. Continued attention to this field is essential to enhance child well-being globally.

## CONCLUSIONS

This study employed scientometric methods to analyze global research on children’s exposure to secondhand smoke and its effects. The findings reveal several key trends. First, the scale of research in this area has grown significantly in recent decades. Second, a select group of journals in the field of substance abuse have emerged as the primary outlets for publishing related studies. Third, the United States leads in both productivity and influence, followed by the United Kingdom and China. The research teams led by Ashley L. Merianos and E. Melinda Mahabee-Gittens have made substantial contributions to the academic output on childhood secondhand smoke exposure. Fourth, there is a clear trend toward increasing interdisciplinary collaboration in this field. Finally, the impact of secondhand smoke exposure on children’s behavior and mental health has become a prominent area of research.

## Supplementary Material



## Data Availability

The data supporting this research are available from the authors on reasonable request.
