# Supplementary material for: Trends of secondhand smoke exposure among children: A scientometric analysis
Source: Tob Induc Dis. 2025 Mar 20;23:10.18332/tid/202017. doi: 10.18332/tid/202017 (PMC11924373; doi:10.18332/tid/202017)
Supplement: Supplementary file 1 [file TID-23-38-s1.pdf]

Supplementary

Supplementary table 1. Search terms and inclusion criteria.

Domain	Terms
Second-hand smoke	(passive or second-hand or involuntary or environment*) And (smok* or tobacco or cigarette)<br>(smok* or tobacco or cigarette or environment*) And (exposure or pollution)
Children and Adolescents	Child or Children or youth* or teenager* or adolescent* or student* or infant*
Age	0-18
Document Type	Article or Review-Article
Language	English

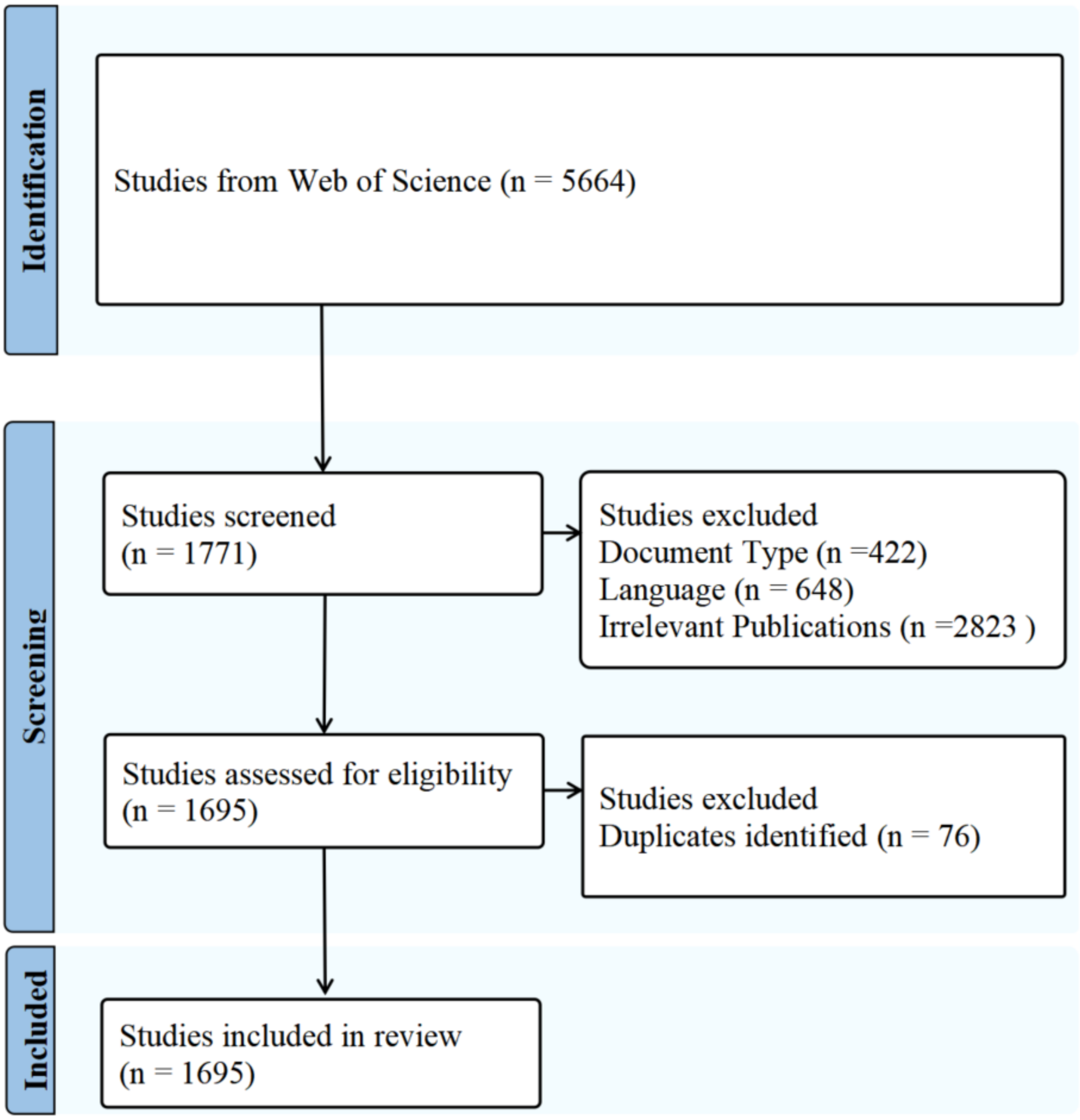

Supplementary figure 1. The Preferred Reporting Items for Systematic Reviews (PRISMA) flow diagram

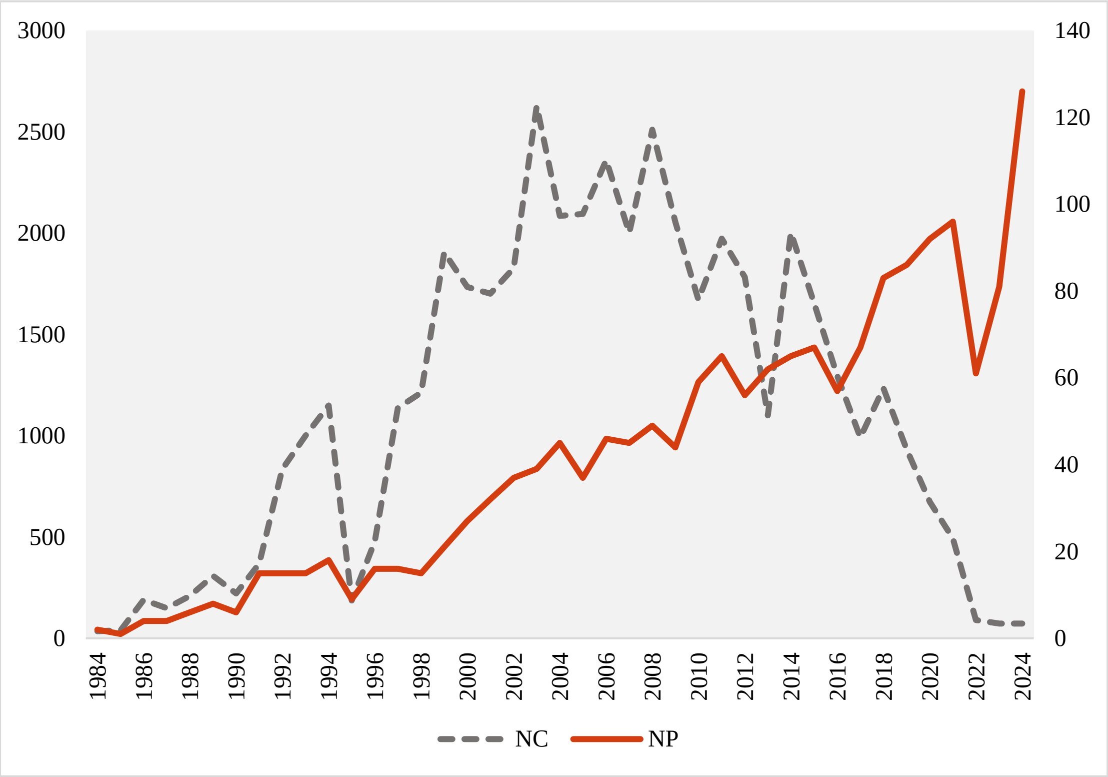

Supplementary figure 2. Annual output of children secondhand smoke research (1984-2024)

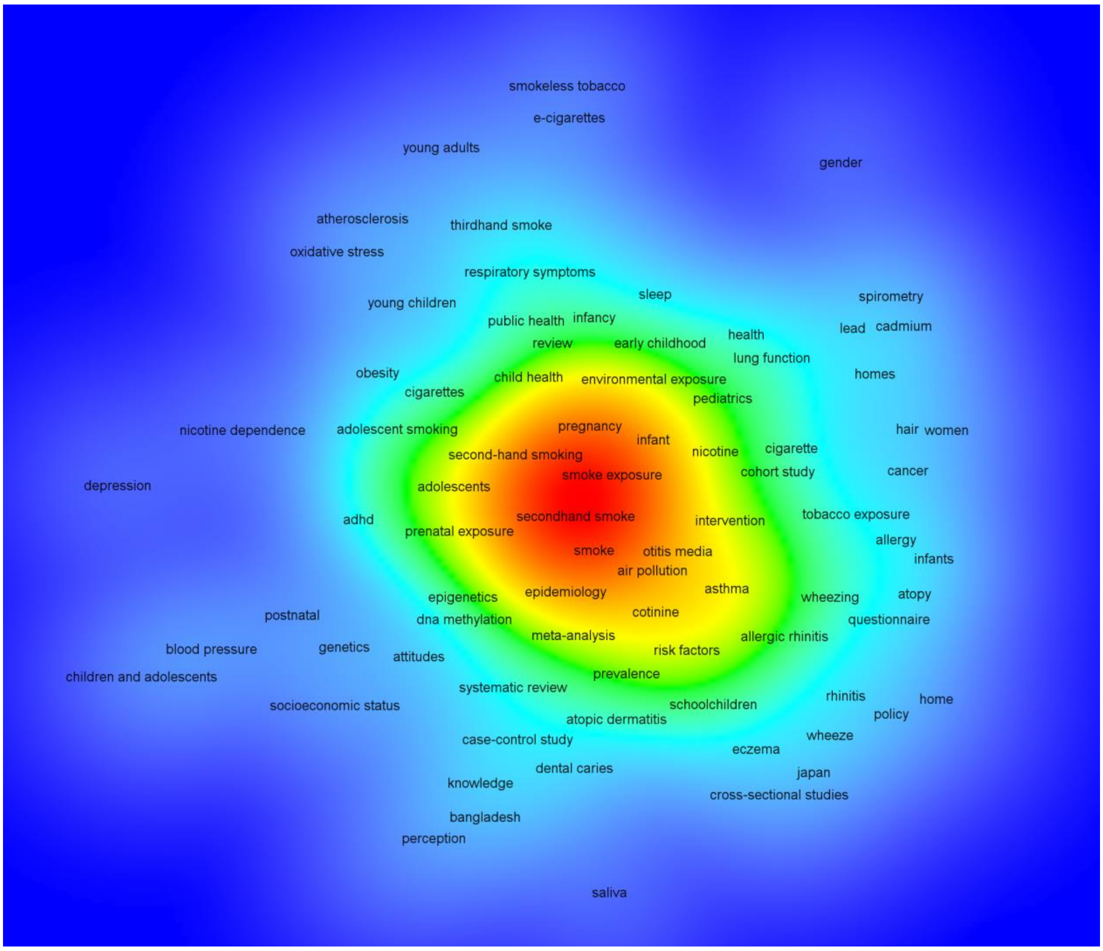

Supplementary figure 3. Keyword co-occurrence. The font size and colour of a keyword indicates how often it appears, and the larger the font size and darker the colour, the more often it appears. (1984-2024)

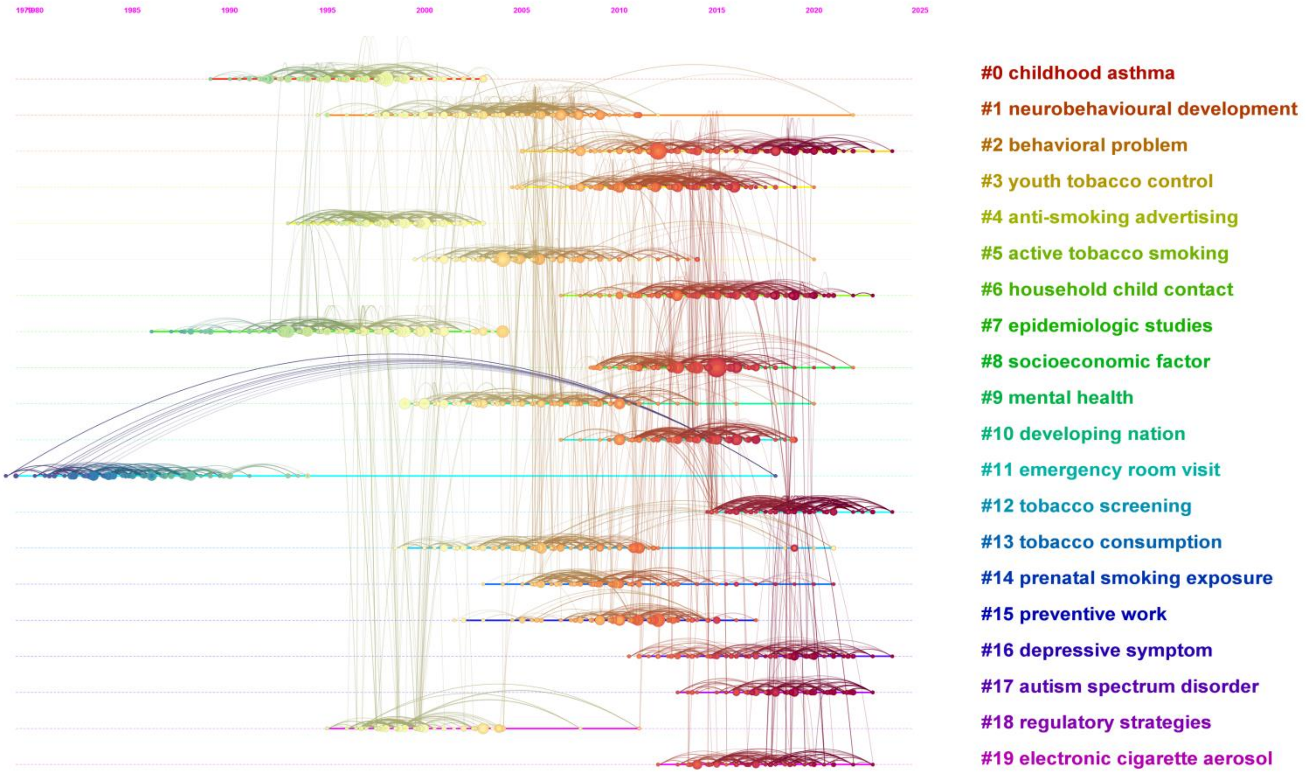

Supplementary figure 4. Timeline view of co-citation cluster. The timeline visualizes the largest 20 clusters of cited literature along the horizontal timeline. Each cluster is arranged vertically in descending order of size, with nodes within the cluster arranged chronologically on the same horizontal line. Colored curves represent co-citation links added in the corresponding colored years. The closer the nodes are to the right, the more recent the topic of a cluster. (1984-2024)

© 2025 Lu H. et al.
